# High nasopharyngeal SARS-CoV-2 load and delayed clearance in hospitalized patients with blood autoantibodies neutralizing type I interferons

**DOI:** 10.1093/infdis/jiag172

**Published:** 2026-09-23

**Authors:** Astrid Marchal, Thibault Kerdiles, Aglaé Perrin, Adrian Gervais, Paul Bastard, Lucy Bizien, Guillaume Lingas, Clément Massonaud, Maude Bouscambert-Duchamp, Alexandre Gaymard, Nathan Peiffer-Smadja, France Mentre, Jérémie Guedj, Laurent Abel, Jean-Laurent Casanova, Anne Puel, Maya Hites, Florence Ader, Julie Bertrand, Aurélie Cobat, the DisCoVeRy Study Group

**Affiliations:** 1 Laboratory of Human Genetics of Infectious Diseases, Necker Branch, Institut National de la Santé et de la Recherche Médicale (INSERM) U1163, Necker Hospital for Sick Children, Paris, France, EU; 2 Imagine Institute, Paris Cité University, Paris, France, EU; 3 AP-HP, Département des Maladies Infectieuses et Tropicales, Hôpital Saint-Louis, Lariboisière, Paris, France, EU; 4 Faculté de Médecine, Sorbonne Université, Paris, France, EU; 5 Université Paris Cité, IAME, INSERM, Paris, France, EU; 6 St. Giles Laboratory of Human Genetics of Infectious Diseases, Rockefeller Branch, The Rockefeller University, New York, NY, USA; 7 Pediatric Hematology-Immunology and Rheumatology Unit, Necker Hospital for Sick Children, Assistance Publique-Hôpitaux de Paris (AP-HP), Paris, France, EU; 8 Department of Epidemiology, Biostatistics and Clinical Research, Hospital Bichat, APHP, Paris, France;; 9 Laboratoire de Virologie, Institut des Agents Infectieux de Lyon, Centre National de Référence des Virus Respiratoires France Sud, Hospices Civils de Lyon, Lyon, France, EU; 10 Laboratoire VirPath, Centre International de Recherche en Infectiologie (CIRI), Inserm 1111, CNRS UMR5308, École Normale Supérieure de Lyon, UCBL, Lyon, France, EU; 11 AP-HP, Hôpital Bichat, Service de Maladies Infectieuses et Tropicales, Paris, France, EU; 12 National Institute for Health Research, Health Protection Research Unit in Healthcare Associated Infections and Antimicrobial Resistance, Imperial College London, London, UK; 13 Department of Pediatrics, Necker Hospital for Sick Children, Paris, France, EU; 14 Howard Hughes Medical Institute, New York, NY, USA; 15 Clinic of Infectious Diseases, Hôpital Universitaire de Bruxelles, Bruxelles, Belgium, EU; 16 Département des Maladies infectieuses et tropicales, Hospices Civils de Lyon, Lyon, France, EU; 17 Centre International de Recherche en Infectiologie (CIRI), Inserm 1111, Université Claude Bernard Lyon 1, CNRS, UMR5308, École Normale Supérieure de Lyon, Univ Lyon, Lyon, France, EU

**Keywords:** SARS-CoV-2, anti-type I interferon autoantibodies, viral load

## Abstract

Autoantibodies neutralizing type I interferon (AAN-I-IFNs) underlie life-threatening COVID-19. In hospitalized COVID-19 patients of the DisCoVeRy trial (NCT-04315948), AAN-I-IFNs were associated with higher nasopharyngeal viral load at inclusion (p=1.4×10^−3^) and delayed viral clearance (p=0.013). Blood AAN-I-IFNs, high viral load and delayed clearance were also independent risk factors for critical COVID-19.

## SUMMARY

In COVID-19 patients of the DisCoVeRy trial, Autoantibodies neutralizing type I interferon (AAN-I-IFNs) were associated with higher nasopharyngeal viral load at inclusion and delayed clearance. AAN-I-IFNs, high viral load and delayed clearance were also independent risk factors for critical COVID-19.

## INTRODUCTION

Interindividual clinical variability following SARS-CoV-2 infection is vast, ranging from asymptomatic infections to life-threatening respiratory failure [1]. Advanced age is the strongest epidemiological risk factor for hypoxemic COVID-19 pneumonia, but male sex and several comorbidities ( e.g. obesity, diabetes, cardiovascular diseases) were also associated with poorer outcomes [2]. Studies of SARS-CoV-2 viral kinetics provide important insights into both disease progression and transmission potential [2,3]. Indeed, viral loads in the respiratory tract, particularly beyond the first week of illness, are higher in patients with severe disease than in those with mild COVID-19 [4,5]. Prolonged viral shedding is also more frequent in immunocompromised individuals, older individuals and men [6], mirroring epidemiological trends of disease severity. The DisCoVeRy trial is a randomized, phase III platform clinical trial (NCT-04315948; EudraCT2020–000936-23), evaluating antiviral drugs in hospitalized COVID-19 patients [3,7].

Type I interferons (IFNs) are essential for antiviral immunity, particularly during early SARS-CoV-2 infection [8]. Autoantibodies neutralizing type I interferons (AAN-I-IFNs) account for ≥15% of severe COVID-19 cases and represent one of the most common predictor of life-threatening COVID-19, after advanced age [9]. These autoantibodies precede infection and impair type I interferon immunity throughout illness. These findings have been replicated worldwide [9]. AAN-I-IFNs have been found in blood, nasal secretions, tracheal aspirates, alveolar lavages [1,9], and cerebro-spinal fluid [10]. They also underlie severe cases of other viral illnesses such as influenza pneumonia, Middle East respiratory syndrome, and several arboviral encephalitis [10]. Their impact on clinical outcomes is well established, but their effects on SARS-CoV-2 viral kinetics in the upper respiratory tract, including nasopharyngeal viral load and infected cell elimination rate, remain unclear [11,12]. Here, we investigated the relationship between AAN-I-IFNs and SARS-CoV-2 viral kinetics in patients enrolled in the DisCoVeRy trial. Nasopharyngeal viral load has been closely monitored in these patients, and a mathematical model was fitted to reconstruct their viral kinetic profiles. Remdesivir reduced viral production by infected cells 2-fold in this model [3].

## METHODS

### Patients:

We analyzed hospitalized COVID-19 patients enrolled in the DisCoVeRy trial between March 2020 and January 2021, randomized to receive either standard of care (SoC) alone or SoC with remdesivir. We defined patients with critical disease as those requiring Intensive Care Unit admission, and/or experiencing Acute Respiratory Distress Syndrome and/or dying during the study. All participants provided written informed consent.

### Viral load measurements and kinetics

Nasopharyngeal swabs (NPS) were collected at randomization and various timepoints thereafter (up to 29 ±3 days). Viral loads were measured by rtRT-PCR and normalized to the total number of cells in NPS (log_10_ of RNA copies per 10^4^ cells). Normalized viral loads < 1 were considered below the limit of detection (LoD) and set to 1. A nonlinear mixed-effect model was previously used to reconstruct viral kinetics using the full viral load profiles (Supplementary methods) [3]. Here, we focused on two phenotypes: i) empirical Bayes estimate of the infected cell loss rate δ from [3]; and ii) normalized viral load at randomization (VLr).

### Autoantibodies neutralizing type I interferons

We assessed the ability of plasma samples collected at randomization to neutralize either high (10ng/ml) or low (100pg/ml) concentrations of IFN-α2 or IFN-ω by luciferase assays, as previously described [13] (Supplementary methods). For each interferon and dose, samples with induction levels below 15% of the median of non-neutralizing samples were classified as neutralizing [13]. Based on the results of the four luciferase assays, we defined three patient groups: i) neutralizing high doses of both IFN-α and IFN-ω (AAN-I-IFNs-10 carriers); ii) neutralizing low doses of IFN-α and/or IFN-ω (AAN-I-IFNs-100 carriers, including AAN-I-IFNs-10); and iii) showing no neutralizing activity at any IFNs and doses tested (non-carriers).

### Statistical analysis

Associations between δ and AAN-I-IFNs were assessed using linear regression models (A) with the individual empirical Bayes estimate (EBE) of the δ random effect ($\eta_{\delta }$) as the dependent variable. For interpretability, mean δ values are reported for AAN-I-IFNs carriers and non-carriers. Linear regression on EBE is standard practice when shrinkage is modest, as observed here (19.9% based on the standard deviation ratio and 35.8% based on the variance ratio) [14,15]. Effect heterogeneity by treatment arm was assessed via an AAN-I-IFNs × treatment arm interaction term. Associations between VLr and AAN-I-IFNs were assessed using linear regression models (B), with effect modification by time since symptom onset (TSO < or ≥7 days) explored through stratified analyses. Associations between COVID-19 severity and AAN-I-IFNs, δ or VLr were assessed using logistic regression models (C). Covariates adjustment was outcome-specific. Models (A) were adjusted for sex only, as age was already accounted for in the viral dynamic model and δ is not dependent on sampling time. Models (B) were adjusted for sex, age (+/− 65 years old) and TSO. Models (C) were adjusted for sex and age, with TSO included only in analyses involving VLr. Mediation analyses were conducted using the mediation R package.

## RESULTS

### AAN-I-IFNs in DisCoVeRy patients:

Patients were recruited for the DisCoVeRy trial between March 2020 and January 2021. They form a homogeneous cohort of unvaccinated hospitalized patients infected with pre-Omicron variants. We included 432 patients with previously modelled viral kinetic profiles [3] and plasma samples collected at randomization for neutralization assays (Figure S1). This subset was representative of the full cohort (n=830, Table S1). Patients had a mean (± standard error of mean [SEM]) age of 63 (±0.6) years at inclusion and 68% were male (Table S1). Treatment arms were equally represented in this subset, with 54% of patients receiving SoC+remdesivir. Dexamethasone was added to SoC during the trial and 49% of patients received corticosteroids. Most patients (430, 99.5%) required oxygen supplementation during hospitalization and about half (53%) developed critical pneumonia. We detected autoantibodies neutralizing 100pg/mL of IFN-α and/or IFN-ω (AAN-I-IFNs-100) in 53/432 (12.3% [95% CI: 4.9–9.8%]) patients, including 37 (8.6% [95% CI: 6.3–11.6%]) patients with autoantibodies neutralizing 10ng/ml of IFN-α and/or IFN-ω (Tables S2-3). Most carriers neutralized both IFN-α and IFN-ω. Specifically, 30 (6.9% [95% CI: 6.1–11.3]) had autoantibodies neutralizing 10ng/mL of both cytokines (AAN-I-IFNs-10). For further analysis, we considered two definitions of autoantibodies: a stringent definition (AAN-I-IFNs-10) and a more inclusive definition (AAN-I-IFNs-100), as explained above. We compared baseline demographics and comorbidities between AAN-I-IFNs-10 carriers, AAN-I-IFNs-100 carriers and non-carriers (Supplementary methods, Table S2). Sex was moderately associated with AAN-I-IFNs, with a higher proportion of males among AAN-I-IFNs-100 carriers than non-carriers (81% vs. 66%, p=0.029), consistent with previous reports [8,13].

### AAN-I-IFNs are associated with slower viral clearance and higher viral load at randomization

**Figure 1.**
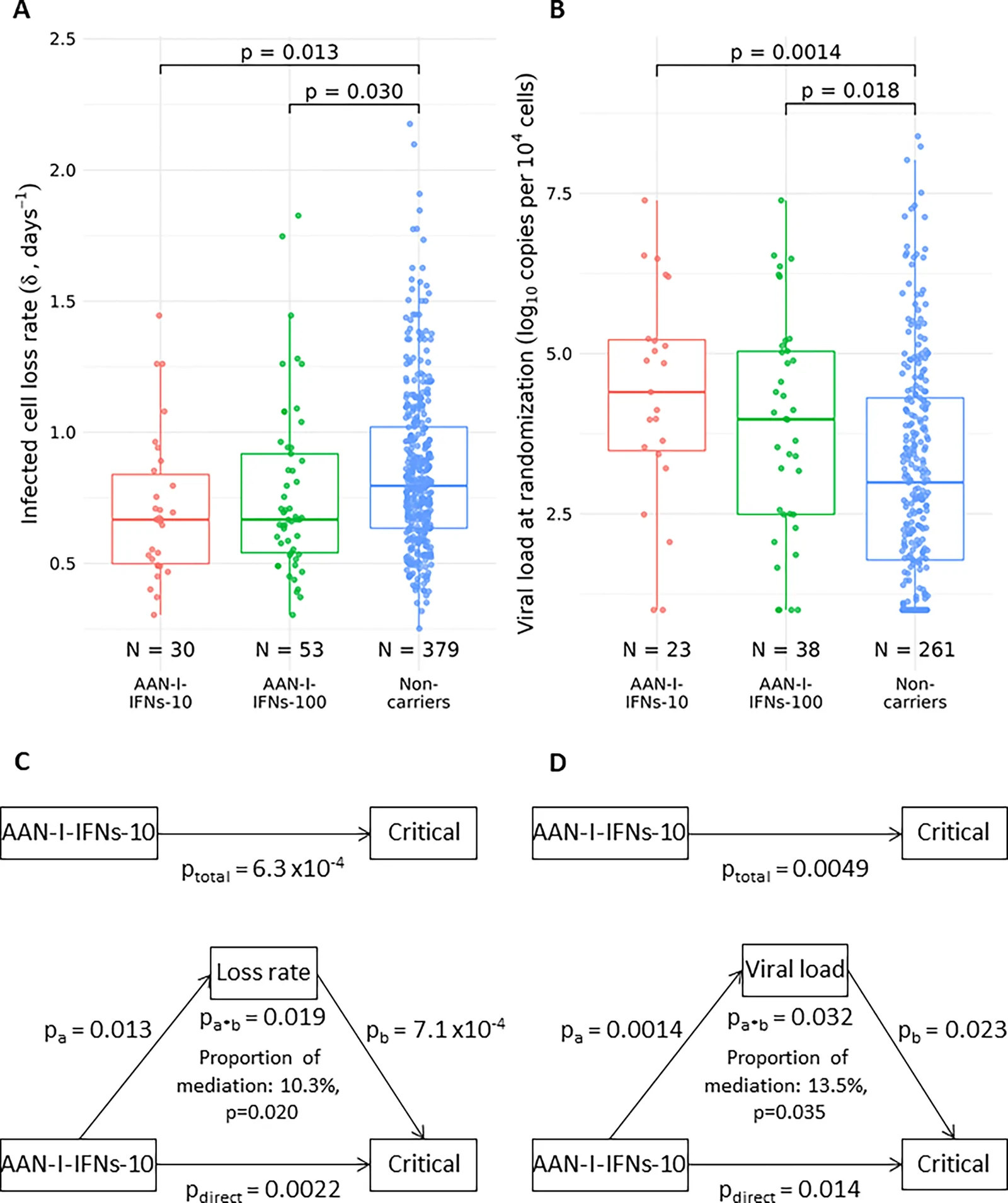
A: Comparison of infected cell loss rates (δ) between carriers of AAN-I-IFNs-10 or AAN-I-IFNs-100 and non-carriers. P-values were obtained from linear regression models of empirical Bayes estimates of the δ random effect ($\eta_{\delta }$) on AAN-I-IFNs-10 or AAN-I-IFNs-100, adjusted for sex. B: Comparison of viral load at randomization (VLr; log_10_ copies per 10^4^ cells) between carriers of AAN-I-IFNs-10 or AAN-I-IFNs-100 and non-carriers. P-values were obtained from linear regression models of VLr on AAN-I-IFNs-10 or AAN-I-IFNs-100, adjusted for age, sex and time since symptom onset. C, D: Path diagrams of the mediation analyses of infected cell loss rate (C; N=432) or viral load at randomization (VLr) (D; N=299) on the relationship between AAN-I-IFNs-10 and critical COVID-19. P_total_ and P_a_ were obtained from the corresponding separate regression models. P_b_ were obtained from the corresponding combined regression models. P_direct_, P_a*b_ and proportion of mediation were derived from the mediation analyses.

A viral dynamics model was previously fitted to full nasopharyngeal viral load profiles, providing each patient with an empirical Bayes estimate of infected cell loss rate (δ) [3]. The mean loss rate (±SEM) was 0.89 (±0.02) days^−1^ in individuals aged <65 years and 0.80 (±0.02) days^−1^ in those aged ≥65 years (Figure S2). Patients with AAN-I-IFNs had significantly lower infected cell elimination rates, with a mean δ (±SEM) of 0.85 (±0.02) days^−1^ for non-carriers, as compared with a mean δ (±SEM) of 0.77 (±0.04) days^−1^ for AAN-I-IFNs-100 carriers (p=0.030) and 0.71 (±0.05) days^−1^ for AAN-I-IFNs-10 carriers (p=0.013) (Figure 1A). Likelihood-ratio-tests comparing viral dynamic models with and without AAN-I-IFN yielded similar conclusions (p_AAN-I-IFNs-100_=0.002 and p_AAN-I-IFNs-10_=0.0004). No significant heterogeneity by treatment arm was observed for the effect of AAN-I-IFNs (p_AAN-I-IFNs-100_=0.61 and p_AAN-I-IFNs-10_=0.86, Figure S3). We then investigated the effect of AAN-I-IFNs on nasopharyngeal viral load measured at randomization (VLr) in a subsample of 299 patients. For 13% of samples, the VLr was below the LoD and was set to 1. The mean VLr (±SEM) was 3.3 (±0.1) log_10_ copies per 10^4^ cells (Figure S4). AAN-I-IFNs-100 were detected in 38 (13%) of these patients, including 23 (8%) who also had AAN-I-IFNs-10. Patients carrying AAN-I-IFNs-100 had significantly higher VLr than non-carriers, with an increase of 0.7 log_10_ copies per 10^4^ cells (p=0.018). The difference was greater in AAN-I-IFNs-10 carriers, with an increase of 1.2 log_10_ copies per 10^4^ cells (p=1.4 ×10^−3^) compared with non-carriers (Figure 1B). Consistent results were obtained when excluding VLr<LoD values and with a left-censored Tobit model (Table S4). Interestingly, the effect was stronger in patients included more than seven days after symptom onset (increase of 1.2 log_10_ copies per 10^4^ cells, p=3.6 ×10^−3^ for AAN-I-IFNs-100 carriers; increase of 1.7 log_10_ copies per 10^4^ cells, p=3.8 ×10^−4^ for AAN-I-IFNs-10 carriers) (Figure S5).

### AAN-I-IFNs and viral kinetics are associated with COVID-19 severity

We then investigated the effect of AAN-I-IFNs-10 and viral kinetics on the risk of critical COVID-19. In models fitted separately for each risk factor, critical COVID-19 was significantly associated with i) the presence of AAN-I-IFNs-10 (p=6.3 ×10^−4^), ii) slower infected cells elimination (p=3.5 ×10^−4^) and iii) higher VLr (p=0.012) (Table S5). The 227 patients with critical pneumonia included 35 (15%) with AAN-I-IFNs-100 and 25 with AAN-I-IFNs-10 (11%), whereas these autoantibodies were detected in 18 (8.8%, p=0.040) and 5 (2.4%, p=4.8 ×10^−4^), respectively, of patients with non-critical disease. In fully adjusted models jointly including AAN-I-IFNs-10 and either δ or VLr, all effects remained significant on critical COVID-19 (Tables S6 and S7). We further assessed the combined effect of AAN-I-IFNs-10 and viral kinetics through mediation analyses, which decompose the total effect of a given risk factor (here, AAN-I-IFNs) on a clinical outcome (here, critical COVID-19) into a direct effect and an indirect effect through a mediator variable (here, viral parameters) [16]. Considering infected cell loss rate as the mediator, 10.3% (95% CI: 1.2–32%) of the total effect of AAN-I-IFNs-10 on COVID-19 severity was mediated (p=0.020), with a significant remaining direct effect of AAN-I-IFNs-10 (p=0.0022, Figure 1C). Considering VLr as the mediator, 13.5 % (95% CI: 6.4–50%) of this effect was mediated (p=0.035), with a significant remaining direct effect (p=0.014, Figure 1D). In conclusion, the effects of AAN-I-IFNs and viral kinetics on COVID-19 severity are mostly independent.

## DISCUSSION

Consistent with previous reports [8,13], we detected AAN-I-IFNs in 12% of 432 hospitalized COVID-19 patients from the DisCoVeRy trial. Most neutralized both IFN-α and IFN-ω, with 6.9% neutralizing both interferons at 10ng/ml. Viral clearance was slower in patients with AAN-I-IFNs than in non-carriers, with a significantly lower infected cell loss rate, as estimated with a mathematical model used to fit viral load profiles [3]. Interestingly, some non-carriers had extreme viral outcomes values potentially explained by other conditions affecting type I IFNs, such as genetic causes (e.g. 1% of male <60 years with critical COVID-19 are known to have TLR7 deficiency [1]). Our results are consistent with the longer time to viral clearance observed by Abers et al. in AAN-I-IFNs-10 carriers (mean 24 days) than in non-carriers (mean 15 days in the overall cohort) [11]. Patients with AAN-I-IFNs also had a significantly higher VLr, with a stronger effect in patients with AAN-I-IFNs-10 than in those with AAN-I-IFNs-100, and an even stronger effect in patients included more than seven days after symptom onset. This is consistent with the capacity of AAN-I-IFNs to neutralize interferons, thereby facilitating viral replication.

As both AAN-I-IFNs and viral kinetics are associated with COVID-19 severity, we investigated their combined effect and quantified the effect of pre-existing AAN-I-IFNs mediated by VLr or by the estimated rate of infected cell loss. Importantly, the infected cell loss rate is a dynamic parameter that reflects host-virus interactions over time and is therefore particularly relevant for mediation analysis. Nevertheless, mediation analyses revealed that 90% of the effect of AAN-I-IFNs on COVID-19 severity was independent of these two viral parameters. This suggests that the effect of AAN-I-IFNs is only partly explained by their influence on viral replication in the upper respiratory tract. Several non–mutually exclusive mechanisms may account for this finding. COVID-19 severity is primarily driven by lower respiratory tract involvement, and nasopharyngeal viral load may represent only an indirect proxy of the pathogenic processes. Type I interferons regulate multiple aspects of antiviral immunity beyond direct viral control, including innate immune cell activation and shaping of adaptive immune responses[17,18]. Finally, AAN-I-IFNs predispose to herpesvirus reactivations, which have been associated with worse clinical outcomes but are largely independent of SARS-CoV-2 nasopharyngeal viral kinetics[19].

## Supplementary Material

Fig S1

Fig S2

Fig S3

Fig S4

Fig S5

Table S1

Table S2

Table S3

Table S4

Table S5

Table S6

Table S7

Methods S

Supplementary data contain supplementary methods, 5 supplementary figures, and 7 supplementary tables.

## Data Availability

Data not publicly available.
