# Supplementary material for: High nasopharyngeal SARS-CoV-2 load and delayed clearance in hospitalized patients with blood autoantibodies neutralizing type I interferons
Source: J Infect Dis. Author manuscript; Available in PMC 2026 Aug 12. (PMC13460755; doi:10.1093/infdis/jiag172)
Supplement: Supplementary file 3 [file NIHMS2185128-supplement-Fig_S3.docx]

***Figure S3:*** *Comparison of infected cell loss rate (δ, days^-1^) between carriers of AAN-I-IFNs-10 or AAN-I-IFNs-100 and non-carriers, stratified by treatment arm.*
