# Supplementary material for: High nasopharyngeal SARS-CoV-2 load and delayed clearance in hospitalized patients with blood autoantibodies neutralizing type I interferons
Source: J Infect Dis. Author manuscript; Available in PMC 2026 Aug 12. (PMC13460755; doi:10.1093/infdis/jiag172)
Supplement: Supplementary file 4 [file NIHMS2185128-supplement-Fig_S4.docx]

***Figure S4:*** *Distribution of the viral load (log_10_ copies per 10^4^ cells) at randomization (VLr). The vertical dotted line represents the mean VLr value in the sample.*
