# Supplementary material for: High nasopharyngeal SARS-CoV-2 load and delayed clearance in hospitalized patients with blood autoantibodies neutralizing type I interferons
Source: J Infect Dis. Author manuscript; Available in PMC 2026 Aug 12. (PMC13460755; doi:10.1093/infdis/jiag172)
Supplement: Supplementary file 6 [file NIHMS2185128-supplement-Methods_S.docx]

**Supplementary data**

**Supplementary Methods:**

*Viral load measurements*

As detailed in Ader F et al. [1], determination of the viral load was done , on nasopharyngeal swabs specimens collected with validated devices containing flocked swabs and virus transport medium, at randomization (day 1) and on days 3, 5, 8, 11, 15 ±2 and 29 ±3 after randomization. RNA extraction was performed on the EMAG platform (bioMerieux, Marcy-l'Étoile, France) following manufacturer’s instructions. The SARS-CoV-2 load was measured by quantitative rtRT-PCR, according to a scale of calibrated in-house plasmid, using the real time RT-PCR RdRp-IP4 developed by the Institut Pasteur (Paris, France) [2]. The amplification protocol was developed using QuantStudio 5 rtRT-PCR Systems (Thermo Fisher Scientific, Waltham, MA, USA). The number of cells in nasopharyngeal sample was further determined using the CELL Control r-gene kit (Argene-BioMérieux, Marcy-l'Étoile, France), which provides a quantified plasmid with the HPRT1 housekeeping gene [3], for quality control purpose and normalization of the viral load measurements. Several studies have shown that epithelial cells are predominant, but immune cells are also present in variable proportions [4–6]. If cell quantification was less than 500 cells per reaction, the quality of the sample was considered too low to be measured. The normalized SARS-CoV-2 load in log_10_ copies per 10^4^ cells was computed by dividing the viral load by the number of cells. Standardized viral loads less than 1 log_10_ copies per 10^4^ cells were considered below the limit of detection (LoD) and set to 1 [7]. Only viral load at randomization (VLr) was considered in this study.

*Viral dynamics model*

A target-cell limited model with fixed effects was fitted to the viral load data (N=684), to reconstruct the viral kinetics from the time of infection until viral clearance, as previously described [7]. Fixed effects and inter-individual variance of the random effects were estimated with the stochastic approximation expectation–maximization (SAEM) algorithm. We focused here on the infected cell loss rate per day (δ), which was modelled assuming a log-normal distribution with an effect of age:

$$\log(\delta_{i})= \mu_{\delta}+\beta_{\mathrm{age}}\times I_{\{\mathrm{age}_{i}\geq65\}} + \eta_{\delta_{i}}$$

with:	$\eta_{\delta} \sim N(0,\omega_{\delta})$

For statistical tests, we used the empirical Bayes random effect estimates ($\eta_{\delta_{i}}$) of the infected cell loss rate, which are normally distributed and correspond to the departure of an individual from the mean value for the whole cohort after accounting for the effect of age. A negative or positive $\eta_{\delta_{i}}$ indicates that individual *i* eliminates infected cells slower or faster, respectively, than the mean value for the cohort. The estimates of infected cell loss rate took into account remdesivir effect, as described elsewhere [7].

*Autoantibodies neutralizing type I interferons*

We assessed the ability of plasma samples collected at randomization to neutralize either high (10 ng/ml) or low (100 pg/ml) concentrations of IFN-α2 or IFN-ω, byluciferase assays, as previously described [8]. We did not assess the neutralization of IFN-β. Briefly, we transfected HEK293T cells with a plasmid containing the firefly luciferase gene under the control of the human ISRE promoter and a plasmid constitutively expressing the *Renilla* luciferase gene for normalization. Cells were stimulated with either IFN-α2 or IFN-ω at a concentration of 10 ng/ml or 100 pg/ml, in the presence of 10% healthy control or patient plasma. Luminescence was measured after cell lysis. For each interferon and dose, the firefly Luciferase to *Renilla* luciferase luminescence intensity ratio (L/R) was further normalized against a control value to account for day-to-day variability and expressed as a percentage. This control value was defined as the median L/R for all individuals screened on the same day, after the removal of possible neutralizing samples, i.e. samples with L/R < 20 at 100 pg/mL or L/R < 50 at 10 ng/mL. As previously described [8–12], samples with induction levels below 15% of the control value for a given interferon and dose were classified as neutralizing for the corresponding interferon and dose. This threshold was empirically determined to provide optimal separation between positive and negative controls during assay development, enabling robust detection of strongly neutralizing plasmas while minimizing false positives. We summarized the results of the four luciferase assays into two categorical variables: one indicating the ability to neutralize high doses (10 ng/ml) of both IFN-α2 and IFN-ω (AAN-I-IFNs-10, which has been identified as the combination providing the strongest risk for critical COVID-19 [8,13]), and another indicating the ability to neutralize low doses (100 pg/ml of IFN-α2 and/or IFN-ω (AAN-I-IFNs-100). All patients carrying AAN-I-IFNs-10 are included in the AAN-I-IFNs-100 carriers’ category. Samples showing no neutralizing activity across all interferons and concentrations tested were considered non-neutralizing and used as controls.

*Statistical analysis*

Patients included in this study were compared with DisCoVeRy patients not included, carriers of AAN-I-IFNs-100 were compared with non-carriers, and carriers of AAN-I-IFNs-10 were compared with non-carriers in Student’s t-tests for continuous variables (i.e. age, viral load and infected cell loss rate) or in Fisher’s exact tests for nominal variables (i.e. sex, comorbidities, severity, treatment).

The association between the infected cell loss rate estimate (δ) and the presence of AAN-I-IFNs-10 or AAN-I-IFNs-100 was investigated by linear regression with the empirical Bayes estimate (EBE) of the δ random effect ($\eta_{\delta}$) as dependent variable. This regression was adjusted for sex only, as age and time since symptom onset are already taken into account in $\eta_{\delta}$. The homogeneity of the effect of AAN-I-IFNs across treatment arms was assessed through an interaction term between AAN-I-IFNs and treatment arm.

The association between nasopharyngeal viral load at randomization (VLr) and the presence of AAN-I-IFNs-10 or AAN-I-IFNs-100 was assessed by linear regression with adjustment for age in two categories (< 65 versus ≥ 65 years old), sex, and time since symptoms onset (TSO) at randomization in two categories (≤ 7 versus > 7 days). We choose this cutoff of seven days after symptom onset to ensure consistency with previous analysis [1,7]. This association was also tested using the same linear regression model excluding values below the LoD, and using a left-censored Tobit model, as implemented in the AER package of R software. The heterogeneity of the effect of AAN-I-IFNs according to TSO was assessed by stratified analyses (TSO ≤ 7 or > 7 days).

The risk of critical COVID-19 conferred by AAN-I-IFNs-10 relative to non-carrier status or AAN-I-IFNs-100 relative to non-carrier status was estimated by logistic regression with adjustment for age in two categories and sex. The association between critical COVID-19 and infected cell loss rate estimates was assessed by logistic regression on the random effect ($\eta_{\delta}$) with adjustment for age (in two categories) and sex in a separate model, and in a fully adjusted model including AAN-I-IFNs-10. The association between critical COVID-19 and VLr was assessed by logistic regression with adjustment for age in two categories, sex and TSO in two categories in a separate model, and in a fully adjusted model including AAN-I-IFNs-10. All linear and logistic regressions were performed with the glm function of R software.

Mediation analyses were conducted using the mediation R package [14], which implements the method developed by Imai, Keele and Yamamoto within a counterfactual causal inference framework [15]. We used nonparametric bootstrapping with 10,000 simulations. The key assumption of this approach is sequential ignorability, which requires the absence of unmeasured confounding of the exposure–outcome and mediator–outcome relationships, conditional on the included covariates. As this assumption is not directly testable, we performed a sensitivity analysis to assess the robustness of the mediation results to potential violations. This analysis indicated that the direct effect of AAN-I-IFNs on COVID-19 severity was robust (a high residual correlation between the mediator and the outcome of $\rho_{\mathrm{critical}}$=0.8 or -0.7, when considering infected cell loss rate or VLr as the mediator, respectively, would be needed to render this effect non-significant), whereas the mediated effect through viral load or infected cell loss rate was more sensitive to unmeasured confounding (requiring modest residual correlation of $\rho_{\mathrm{critical}}$=-0.2 and 0.1, respectively, to be non-significant). The observed residual correlations were negligible (ρ=-0.04 and 0.03, respectively). These results are consistent with the association between AAN-I-IFNs and COVID-19 severity being only partially mediated by infected cell loss rate or by viral load.

*References*:

[1]	Ader F, Bouscambert-Duchamp M, Hites M, Peiffer-Smadja N, Poissy J, Belhadi D, et al. Remdesivir plus standard of care versus standard of care alone for the treatment of patients admitted to hospital with COVID-19 (DisCoVeRy): a phase 3, randomised, controlled, open-label trial. The Lancet Infectious Diseases 2022;22:209–21. https://doi.org/10.1016/S1473-3099(21)00485-0.

[2]	Etievant S, Bal A, Escuret V, Brengel-Pesce K, Bouscambert M, Cheynet V, et al. Performance Assessment of SARS-CoV-2 PCR Assays Developed by WHO Referral Laboratories. Journal of Clinical Medicine 2020;9:1871. https://doi.org/10.3390/jcm9061871.

[3]	Santos Bravo M, Nicolás D, Berengua C, Fernandez M, Hurtado JC, Tortajada M, et al. Severe Acute Respiratory Syndrome Coronavirus 2 Normalized Viral Loads and Subgenomic RNA Detection as Tools for Improving Clinical Decision Making and Work Reincorporation. J Infect Dis 2021;224:1325–32. https://doi.org/10.1093/infdis/jiab394.

[4]	Ziegler CGK, Miao VN, Owings AH, Navia AW, Tang Y, Bromley JD, et al. Impaired local intrinsic immunity to SARS-CoV-2 infection in severe COVID-19. Cell 2021;184:4713-4733.e22. https://doi.org/10.1016/j.cell.2021.07.023.

[5]	Walsh JML, Miao VN, Owings AH, Tang Y, Bromley JD, Kazer SW, et al. Variants and vaccines impact nasal immunity over three waves of SARS-CoV-2. Nat Immunol 2025;26:294–307. https://doi.org/10.1038/s41590-024-02052-z.

[6]	Loske J, Röhmel J, Lukassen S, Stricker S, Magalhães VG, Liebig J, et al. Pre-activated antiviral innate immunity in the upper airways controls early SARS-CoV-2 infection in children. Nat Biotechnol 2022;40:319–24. https://doi.org/10.1038/s41587-021-01037-9.

[7]	Lingas G, Néant N, Gaymard A, Belhadi D, Peytavin G, Hites M, et al. Effect of remdesivir on viral dynamics in COVID-19 hospitalized patients: a modelling analysis of the randomized, controlled, open-label DisCoVeRy trial. Journal of Antimicrobial Chemotherapy 2022;77:1404–12. https://doi.org/10.1093/jac/dkac048.

[8]	Bastard P, Gervais A, Le Voyer T, Rosain J, Philippot Q, Manry J, et al. Autoantibodies neutralizing type I IFNs are present in ~4% of uninfected individuals over 70 years old and account for ~20% of COVID-19 deaths. Science Immunology 2021;6:eabl4340. https://doi.org/10.1126/sciimmunol.abl4340.

[9]	Gervais A, Rovida F, Avanzini MA, Croce S, Marchal A, Lin S-C, et al. Autoantibodies neutralizing type I IFNs underlie West Nile virus encephalitis in ∼40% of patients. Journal of Experimental Medicine 2023;220:e20230661. https://doi.org/10.1084/jem.20230661.

[10]	Gervais A, Marchal A, Fortova A, Berankova M, Krbkova L, Pychova M, et al. Autoantibodies neutralizing type I IFNs underlie severe tick-borne encephalitis in ∼10% of patients. Journal of Experimental Medicine 2024;221:e20240637. https://doi.org/10.1084/jem.20240637.

[11]	Gervais A, Bastard P, Bizien L, Delifer C, Tiberghien P, Rodrigo C, et al. Auto-Abs neutralizing type I IFNs in patients with severe Powassan, Usutu, or Ross River virus disease. Journal of Experimental Medicine 2024;221:e20240942. https://doi.org/10.1084/jem.20240942.

[12]	Gervais A, Trespidi F, Ferrari A, Rovida F, Marchal A, Croce S, et al. Autoantibodies neutralizing type I IFNs in 40% of patients with WNV encephalitis in seven new cohorts 2025:2025.08.31.25334556. https://doi.org/10.1101/2025.08.31.25334556.

[13]	Manry J, Bastard P, Gervais A, Le Voyer T, Rosain J, Philippot Q, et al. The risk of COVID-19 death is much greater and age dependent with type I IFN autoantibodies. Proceedings of the National Academy of Sciences 2022;119:e2200413119. https://doi.org/10.1073/pnas.2200413119.

[14]	Tingley D, Yamamoto T, Hirose K, Keele L, Imai K. mediation: R Package for Causal Mediation Analysis. Journal of Statistical Software 2014;59:1–38. https://doi.org/10.18637/jss.v059.i05.

[15]	Imai K, Keele L, Tingley D. A general approach to causal mediation analysis. Psychological Methods 2010;15:309–34. https://doi.org/10.1037/a0020761.

**Supplementary figures:**

***Figure S1.*** *Flow chart of the study*

***Figure S2:*** *Distribution of infected cell loss rate (δ, days^-1^), stratified by treatment arm and age.*

***Figure S3:*** *Comparison of infected cell loss rate (δ, days^-1^) between carriers of AAN-I-IFNs-10 or AAN-I-IFNs-100 and non-carriers, stratified by treatment arm.*

***Figure S4:*** *Distribution of the viral load (log_10_ copies per 10^4^ cells) at randomization (VLr). The vertical dotted line represents the mean VLr value in the sample.*

***Figure S5:*** *Comparison of viral load (log_10_ copies per 10^4^ cells) at randomization between carriers of AAN-I-IFNs-10 or AAN-I-IFNs-100 and non-carriers, stratified by time since symptom onset (days).*

**Supplementary tables:**

***Table S1:*** *Characteristics of DisCoVeRy patients. Results are expressed as N (%) or median (IQR1-IQR3), mean (± standard error of mean [SEM]). Patients included in this study were compared with non-included patients in Student’s t-tests for continuous variables (i.e., age) or in Fisher’s exact tests for nominal variables (i.e., sex, comorbidities, severity, treatment). P value < 5.6 x10^‑3^ was considered as statistically significant after Bonferroni correction.*

|  | **Patients in this study (N=432)** | **Remaining patients (N=398)** | ***P value*** |
| --- | --- | --- | --- |
| *Baseline and clinical characteristics* | | | |
| Age (median (IQR),  mean (±SEM)) | 64 (54-73),  63.2 (±0.6) | 63 (55-73),  63.5 (±0.7) | *0.78* |
| Gender  - M  - F | 294 (68%)  138 (32%) | 283 (71%)  115 (29%) | *0.37* |
| Obesity  - BMI >30  - BMI <30 | 150 (35%)  276 (65%) | 129 (33%)  265 (67%) | *0.46* |
| Chronic cardiac disease  - Yes  - No | 115 (27%)  317 (73%) | 114 (29%)  280 (71%) | *0.48* |
| Diabetes  - Yes  - No | 105 (48%)  327 (52%) | 114 (48%)  281 (52%) | *0.16* |
| Auto-inflammatory diseases  - Yes  - No | 25 (6%)  406 (94%) | 16 (4%)  378 (96%) | *0.27* |
| *COVID-19 severity and treatment* | | | |
| Severity  - Critical COVID-19  - Non-critical | 227 (53%)  205 (47%) | 238 (60%)  160 (40%) | *0.036* |
| Treatment:  - SoC+remdesivir  - SoC | 232 (54%)  200 (46%) | 180 (45%)  218 (55%) | *0.015* |
| Corticosteroids:  - Yes  - No | 213 (49%)  219 (51%) | 158 (40%)  240 (60%) | *6.4 x10^‑3^* |

***Table S2:*** *Characteristics of DisCoVeRy patients. Results are expressed as N (%) or median (IQR1-IQR3), mean (± standard error of mean [SEM]). Carriers of AAN-I-IFNs-100 were compared with non-carriers, and carriers of AAN-I-IFNs-10 were compared with non-carriers in Student’s t-tests for continuous variables (i.e., age, viral load and infected cell loss rate) or in Fisher’s exact tests for nominal variables (i.e., sex, comorbidities, severity, treatment). P value < 0.05 are in bold.*

|  | **AAN-I-IFNs-10 carriers** | ***P value***  ***AAN-I-IFNs-10*** | **AAN-I-IFNs-100 carriers** | ***P value***  ***AAN-I-IFNs-100*** | **Non-carriers** |
| --- | --- | --- | --- | --- | --- |
| **Patients with plasma sample at randomization and model parameter estimates: N=432** | **30 (6.9%)** |  | **53 (12%)** |  | **379 (88%)** |
| *Baseline and clinical characteristics* | | | | | |
| Age (median (IQR),  mean (±SEM)) | 66 (60-75),  64.3 (±2.4) | *0.68* | 64 (55-73),  63.1 (±1.8) | *0.93* | 64 (54-73),  63.2 (±0.7) |
| Gender  - M  - F | 24 (8.2%)  6 (4.3%) | *0.16* | 43 (15%)  10 (7.2%) | ***0.029*** | 251 (85%)  128 (93%) |
| Obesity  - BMI >30  - BMI <30 | 10 (6.7%)  20 (7.2%) | *1* | 19 (13%)  34 (12%) | *1* | 131 (87%)  242 (88%) |
| Chronic cardiac disease  - Yes  - No | 10 (8.7%)  20 (6.3%) | *0.39* | 18 (16%)  97 (11%) | *0.24* | 97 (84%)  282 (89%) |
| Diabetes  - Yes  - No | 6 (5.7%)  24 (7.3%) | *0.82* | 14 (13%)  39 (12%) | *0.73* | 91 (87%)  188 (88%) |
| Auto-inflammatory diseases  - Yes  - No | 3 (6.7%)  27 (12%) | *0.42* | 3 (12%)  50 (12%) | *1* | 22 (88%)  356 (88%) |
| *COVID-19 severity and treatment* | | | | | |
| Severity  - Critical COVID-19  - Non-critical | 25 (11%)  5 (2.4%) | ***4.8 x10^‑4^*** | 35 (15%)  18 (8.8%) | ***0.040*** | 192 (85%)  187 (91%) |
| Treatment:  - SoC+remdesivir  - SoC | 11 (4.7%)  19 (9.5%) | *0.059* | 24 (10%)  29 (15%) | *0.24* | 208 (90%)  171 (86%) |
| Corticosteroids:  - Yes  - No | 19 (8.9%)  11 (5.0%) | *0.13* | 32 (15%)  21 (9.6%) | *0.11* | 181 (85%)  198 (90%) |
| *Viral kinetic* | | | | | |
| Time since symptom onset (days) (median (IQR),  mean (±SEM)) | 8.5 (6-10),  8.7 (±0.6) | *0.35* | 8 (6-11),  8.9 (±0.6) | *0.48* | 9 (6-12),  9.3 (±0.2) |
| Infected cell loss rate (days^‑1^)  (median (IQR),  mean (±SEM)) | 0.67  (0.50 – 0.84),  0.71 (±0.05) | ***0.011*** | 0.67  (0.54 – 0.92),  0.77 (±0.04) | *0.072* | 0.80  (0.63 – 1.02),  0.85 (±0.02) |

(Table S1 continues on next page)

(Continued from previous page)

|  | **AAN-I-IFNs-10 carriers** | ***P value***  ***AAN-I-IFNs-10*** | **AAN-I-IFNs-100 carriers** | ***P value***  ***AAN-I-IFNs-100*** | **Non-carriers** |
| --- | --- | --- | --- | --- | --- |
| **Patients with plasma sample and observed viral load at randomization: N=299** | **23 (7.7%)** |  | **38 (13%)** |  | **261 (87%)** |
| *Baseline and clinical characteristics* | | | | | |
| Age (median (IQR),  mean (±SEM)) | 66 (61-75),  65.0 (±2.7) | *0.51* | 63 (55-73),  63.7 (±2.1) | *0.81* | 64 (54-73),  63.2 (±0.8) |
| Gender  - M  - F | 18 (9.2%)  5 (4.9%) | *0.18* | 31 (16%)  7 (6.8%) | ***0.028*** | 165 (84%)  96 (93%) |
| Obesity  - BMI >30  - BMI <30 | 7 (6.5%)  16 (8.5%) | *0.65* | 13 (12%)  25 (13%) | *0.86* | 95 (88%)  163 (87%) |
| Chronic cardiac disease  - Yes  - No | 8 (9.3%)  15 (7.0%) | *0.48* | 13 (15%)  25 (12%) | *0.45* | 73 (85%)  188 (88%) |
| Diabetes  - Yes  - No | 4 (5.6%)  91 (8.4%) | *0.61* | 8 (11%)  30 (13%) | *0.84* | 64 (89%)  197 (87%) |
| Auto-inflammatory diseases  - Yes  - No | 2 (12%)  21 (7.4%) | *0.64* | 2 (12%)  36 (13%) | *1* | 15 (88%)  246 (87%) |
| *COVID-19 severity and treatment* | | | | | |
| Severity  - Critical COVID-19  - Non-critical | 19 (12%)  4 (2.8%) | ***3.9 x10^‑3^*** | 24 (15%)  14 (9.9%) | *0.17* | 133 (85%)  128 (90%) |
| Treatment:  - SoC+remdesivir  - SoC | 8 (4.9%)  15 (11%) | *0.050* | 16 (9.8%)  22 (16%) | *0.12* | 148 (90%)  113 (84%) |
| Corticosteroids:  - Yes  - No | 12 (8.5%)  11 (7.0%) | *0.67* | 20 (14%)  18 (11%) | *0.60* | 122 (85%)  139 (89%) |
| *Viral kinetic* | | | | | |
| Time since symptom onset (days) (median (IQR),  mean (±SEM)) | 8 (6-9),  7.6 (±0.6) | *0.81* | 7.5 (4-9),  7.4 (±0.5) | *0.57* | 7 (5-10),  7.7 (±0.2) |
| Viral load (log_10_ copies per 10^4^ cells) (median (IQR),  mean (±SEM)) | 4.4  (3.5-5.2),  4.3 (±0.4) | ***3.8 x10^‑3^*** | 4.0  (2.5-5.0),  3.8 (±0.3) | ***0.029*** | 3.0  (1.8-4.3),  3.2 (±0.1) |
| Infected cell loss rate (days^‑1^)  (median (IQR),  mean (±SEM)) | 0.66  (0.49 – 0.77),  0.69 (±0.06) | ***0.0025*** | 0.67  (0.56 – 0.88),  0.76 (±0.05) | ***0.041*** | 0.82  (0.66 – 1.09),  0.89 (±0.02) |

***Table S3:*** *Summary of neutralization data of type I IFNs for 432 DisCoVeRy patients.*

| **IFN(s) neutralized** | **N samples neutralizing 10 ng/ml** | **N samples neutralizing 100 pg/ml** |
| --- | --- | --- |
| IFN-α and/or IFN-ω | 37 (8.6%) | 53 (12.3%) * |
| IFN-α and IFN-ω | 30 (6.9%) ** | 36 (8.3%) |
| IFN-α alone | 5 (1.2%) | 8 (1.9%) |
| IFN-ω alone | 1 (0.2%) | 9 (2.1%) |

* AAN-I-IFNs-100 carriers

** AAN-I-IFNs-10 carriers

***Table S4:*** *Association between VLr and AAN-I-IFN status using linear models (with or without VLr < LoD values) and a left-censored Tobit model (considering VLr < LoD as censored values).*

| Model | Phenotype | N cases | N controls | Effect size (Standard Error) | Pvalue |
| --- | --- | --- | --- | --- | --- |
| Linear regression, VLr<LoD set to 1 | AAN-I-IFNs-10 | 23 | 261 | 1.17 (0.37) | 1.4x10^-3^ |
|  | AAN-I-IFNs-100 | 38 | 261 | 0.70 (0.30) | 0.018 |
| Linear regression, VLr<LoD excluded | AAN-I-IFNs-10 | 21 | 227 | 1.16 (0.36) | 1.3 x10^-3^ |
|  | AAN-I-IFNs-100 | 34 | 227 | 0.74 (0.29) | 0.011 |
| Left-censored Tobit regression | AAN-I-IFNs-10 | 23 | 261 | 1.23 (0.41) | 3.0 x10^-3^ |
|  | AAN-I-IFNs-100 | 38 | 261 | 0.74 (0.33) | 0.027 |

***Table S5:*** *Association between COVID-19 severity and infected cell loss rates or viral load at randomization between critical and non-critical patients.*

|  | Mean (±SEM) in critical patients | Mean (±SEM) in non-critical patients | Pvalue |
| --- | --- | --- | --- |
| Infected cell loss rate | 0.78 (±0.02) | 0.91 (±0.02) | 3.5x10^-4^ |
| Viral load at randomization | 3.48 (±0.14) | 3.01 (±0.14) | 0.012 |

***Table S6:*** *Association of AAN-I-IFNs status and* *empirical Bayes estimate of the infected cell loss rate (*$\eta_{\delta}$*) with COVID-19 severity (sample size: n=409), considering them separately or jointly in the adjusted logistic regression models.*

|  | Separate models * | | Full model ^$^ | |
| --- | --- | --- | --- | --- |
|  | Effect size (SE) | Pvalue | Effect size (SE) | Pvalue |
| AAN-I-IFNs-10 | 1.52 (0.50) | 6.3x10^-4^ | 1.39 (0.51) | 2.4x10^-3^ |
| Loss rate random effect | -1.01 (0.29) | 3.5x10^-4^ | -1.01 (0.30) | 7.1x10^-4^ |

* Separate models assessed the associations of AAN-I-IFNs status and, separately, the infected cell loss rate with COVID-19 severity, adjusting on age and sex.

^$^ The full model jointly assessed the association of AAN-I-IFNs and infected cell loss rate with COVID-19 severity, adjusting on age and sex.

***Table S7:*** *Association of AAN-I-IFNs status and VLr with COVID-19 severity (sample size: n=284), considering them separately or jointly in the adjusted logistic regression models.*

|  | Separate models * | | Full model ^$^ | |
| --- | --- | --- | --- | --- |
|  | Effect size (SE) | Pvalue | Effect size (SE) | Pvalue |
| AAN-I-IFNs-10 | 1.43 (0.57) | 4.9x10^-3^ | 1.24 (0.60) | 0.019 |
| VLr | 0.18 (0.07) | 0.012 | 0.17 (0.08) | 0.023 |

* Separate models assessed the associations of AAN-I-IFNs status and, separately, VLr with COVID-19 severity, adjusting on age and sex for AAN-I-IFNs and age, sex and TSO for VLr.

^$^ The full model jointly assessed the association of AAN-I-IFNs and infected cell loss rate with COVID-19 severity, adjusting on age, sex and TSO.
