# Supplementary material for: High nasopharyngeal SARS-CoV-2 load and delayed clearance in hospitalized patients with blood autoantibodies neutralizing type I interferons
Source: J Infect Dis. Author manuscript; Available in PMC 2026 Aug 12. (PMC13460755; doi:10.1093/infdis/jiag172)
Supplement: Supplementary file 7 [file NIHMS2185128-supplement-Table_S1.docx]

***Table S1:*** *Characteristics of DisCoVeRy patients. Results are expressed as N (%) or median (IQR1-IQR3), mean (± standard error of mean [SEM]). Patients included in this study were compared with non-included patients in Student’s t-tests for continuous variables (i.e., age) or in Fisher’s exact tests for nominal variables (i.e., sex, comorbidities, severity, treatment). P value < 5.6 x10^‑3^ was considered as statistically significant after Bonferroni correction.*

|  | **Patients in this study (N=432)** | **Remaining patients (N=398)** | ***P value*** |
| --- | --- | --- | --- |
| *Baseline and clinical characteristics* | | | |
| Age (median (IQR),  mean (±SEM)) | 64 (54-73),  63.2 (±0.6) | 63 (55-73),  63.5 (±0.7) | *0.78* |
| Gender  - M  - F | 294 (68%)  138 (32%) | 283 (71%)  115 (29%) | *0.37* |
| Obesity  - BMI >30  - BMI <30 | 150 (35%)  276 (65%) | 129 (33%)  265 (67%) | *0.46* |
| Chronic cardiac disease  - Yes  - No | 115 (27%)  317 (73%) | 114 (29%)  280 (71%) | *0.48* |
| Diabetes  - Yes  - No | 105 (48%)  327 (52%) | 114 (48%)  281 (52%) | *0.16* |
| Auto-inflammatory diseases  - Yes  - No | 25 (6%)  406 (94%) | 16 (4%)  378 (96%) | *0.27* |
| *COVID-19 severity and treatment* | | | |
| Severity  - Critical COVID-19  - Non-critical | 227 (53%)  205 (47%) | 238 (60%)  160 (40%) | *0.036* |
| Treatment:  - SoC+remdesivir  - SoC | 232 (54%)  200 (46%) | 180 (45%)  218 (55%) | *0.015* |
| Corticosteroids:  - Yes  - No | 213 (49%)  219 (51%) | 158 (40%)  240 (60%) | *6.4 x10^‑3^* |
