# Supplementary material for: High nasopharyngeal SARS-CoV-2 load and delayed clearance in hospitalized patients with blood autoantibodies neutralizing type I interferons
Source: J Infect Dis. Author manuscript; Available in PMC 2026 Aug 12. (PMC13460755; doi:10.1093/infdis/jiag172)
Supplement: Supplementary file 8 [file NIHMS2185128-supplement-Table_S2.docx]

***Table S2:*** *Characteristics of DisCoVeRy patients. Results are expressed as N (%) or median (IQR1-IQR3), mean (± standard error of mean [SEM]). Carriers of AAN-I-IFNs-100 were compared with non-carriers, and carriers of AAN-I-IFNs-10 were compared with non-carriers in Student’s t-tests for continuous variables (i.e., age, viral load and infected cell loss rate) or in Fisher’s exact tests for nominal variables (i.e., sex, comorbidities, severity, treatment). P value < 0.05 are in bold.*

|  | **AAN-I-IFNs-10 carriers** | ***P value***  ***AAN-I-IFNs-10*** | **AAN-I-IFNs-100 carriers** | ***P value***  ***AAN-I-IFNs-100*** | **Non-carriers** |
| --- | --- | --- | --- | --- | --- |
| **Patients with plasma sample at randomization and model parameter estimates: N=432** | **30 (6.9%)** |  | **53 (12%)** |  | **379 (88%)** |
| *Baseline and clinical characteristics* | | | | | |
| Age (median (IQR),  mean (±SEM)) | 66 (60-75),  64.3 (±2.4) | *0.68* | 64 (55-73),  63.1 (±1.8) | *0.93* | 64 (54-73),  63.2 (±0.7) |
| Gender  - M  - F | 24 (8.2%)  6 (4.3%) | *0.16* | 43 (15%)  10 (7.2%) | ***0.029*** | 251 (85%)  128 (93%) |
| Obesity  - BMI >30  - BMI <30 | 10 (6.7%)  20 (7.2%) | *1* | 19 (13%)  34 (12%) | *1* | 131 (87%)  242 (88%) |
| Chronic cardiac disease  - Yes  - No | 10 (8.7%)  20 (6.3%) | *0.39* | 18 (16%)  97 (11%) | *0.24* | 97 (84%)  282 (89%) |
| Diabetes  - Yes  - No | 6 (5.7%)  24 (7.3%) | *0.82* | 14 (13%)  39 (12%) | *0.73* | 91 (87%)  188 (88%) |
| Auto-inflammatory diseases  - Yes  - No | 3 (6.7%)  27 (12%) | *0.42* | 3 (12%)  50 (12%) | *1* | 22 (88%)  356 (88%) |
| *COVID-19 severity and treatment* | | | | | |
| Severity  - Critical COVID-19  - Non-critical | 25 (11%)  5 (2.4%) | ***4.8 x10^‑4^*** | 35 (15%)  18 (8.8%) | ***0.040*** | 192 (85%)  187 (91%) |
| Treatment:  - SoC+remdesivir  - SoC | 11 (4.7%)  19 (9.5%) | *0.059* | 24 (10%)  29 (15%) | *0.24* | 208 (90%)  171 (86%) |
| Corticosteroids:  - Yes  - No | 19 (8.9%)  11 (5.0%) | *0.13* | 32 (15%)  21 (9.6%) | *0.11* | 181 (85%)  198 (90%) |
| *Viral kinetic* | | | | | |
| Time since symptom onset (days) (median (IQR),  mean (±SEM)) | 8.5 (6-10),  8.7 (±0.6) | *0.35* | 8 (6-11),  8.9 (±0.6) | *0.48* | 9 (6-12),  9.3 (±0.2) |
| Infected cell loss rate (days^‑1^)  (median (IQR),  mean (±SEM)) | 0.67  (0.50 – 0.84),  0.71 (±0.05) | ***0.011*** | 0.67  (0.54 – 0.92),  0.77 (±0.04) | *0.072* | 0.80  (0.63 – 1.02),  0.85 (±0.02) |

(Table S1 continues on next page)

(Continued from previous page)

|  | **AAN-I-IFNs-10 carriers** | ***P value***  ***AAN-I-IFNs-10*** | **AAN-I-IFNs-100 carriers** | ***P value***  ***AAN-I-IFNs-100*** | **Non-carriers** |
| --- | --- | --- | --- | --- | --- |
| **Patients with plasma sample and observed viral load at randomization: N=299** | **23 (7.7%)** |  | **38 (13%)** |  | **261 (87%)** |
| *Baseline and clinical characteristics* | | | | | |
| Age (median (IQR),  mean (±SEM)) | 66 (61-75),  65.0 (±2.7) | *0.51* | 63 (55-73),  63.7 (±2.1) | *0.81* | 64 (54-73),  63.2 (±0.8) |
| Gender  - M  - F | 18 (9.2%)  5 (4.9%) | *0.18* | 31 (16%)  7 (6.8%) | ***0.028*** | 165 (84%)  96 (93%) |
| Obesity  - BMI >30  - BMI <30 | 7 (6.5%)  16 (8.5%) | *0.65* | 13 (12%)  25 (13%) | *0.86* | 95 (88%)  163 (87%) |
| Chronic cardiac disease  - Yes  - No | 8 (9.3%)  15 (7.0%) | *0.48* | 13 (15%)  25 (12%) | *0.45* | 73 (85%)  188 (88%) |
| Diabetes  - Yes  - No | 4 (5.6%)  91 (8.4%) | *0.61* | 8 (11%)  30 (13%) | *0.84* | 64 (89%)  197 (87%) |
| Auto-inflammatory diseases  - Yes  - No | 2 (12%)  21 (7.4%) | *0.64* | 2 (12%)  36 (13%) | *1* | 15 (88%)  246 (87%) |
| *COVID-19 severity and treatment* | | | | | |
| Severity  - Critical COVID-19  - Non-critical | 19 (12%)  4 (2.8%) | ***3.9 x10^‑3^*** | 24 (15%)  14 (9.9%) | *0.17* | 133 (85%)  128 (90%) |
| Treatment:  - SoC+remdesivir  - SoC | 8 (4.9%)  15 (11%) | *0.050* | 16 (9.8%)  22 (16%) | *0.12* | 148 (90%)  113 (84%) |
| Corticosteroids:  - Yes  - No | 12 (8.5%)  11 (7.0%) | *0.67* | 20 (14%)  18 (11%) | *0.60* | 122 (85%)  139 (89%) |
| *Viral kinetic* | | | | | |
| Time since symptom onset (days) (median (IQR),  mean (±SEM)) | 8 (6-9),  7.6 (±0.6) | *0.81* | 7.5 (4-9),  7.4 (±0.5) | *0.57* | 7 (5-10),  7.7 (±0.2) |
| Viral load (log_10_ copies per 10^4^ cells) (median (IQR),  mean (±SEM)) | 4.4  (3.5-5.2),  4.3 (±0.4) | ***3.8 x10^‑3^*** | 4.0  (2.5-5.0),  3.8 (±0.3) | ***0.029*** | 3.0  (1.8-4.3),  3.2 (±0.1) |
| Infected cell loss rate (days^‑1^)  (median (IQR),  mean (±SEM)) | 0.66  (0.49 – 0.77),  0.69 (±0.06) | ***0.0025*** | 0.67  (0.56 – 0.88),  0.76 (±0.05) | ***0.041*** | 0.82  (0.66 – 1.09),  0.89 (±0.02) |
