# Supplementary material for: High nasopharyngeal SARS-CoV-2 load and delayed clearance in hospitalized patients with blood autoantibodies neutralizing type I interferons
Source: J Infect Dis. Author manuscript; Available in PMC 2026 Aug 12. (PMC13460755; doi:10.1093/infdis/jiag172)
Supplement: Supplementary file 9 [file NIHMS2185128-supplement-Table_S3.docx]

***Table S3:*** *Summary of neutralization data of type I IFNs for 432 DisCoVeRy patients.*

| **IFN(s) neutralized** | **N samples neutralizing 10 ng/ml** | **N samples neutralizing 100 pg/ml** |
| --- | --- | --- |
| IFN-α and/or IFN-ω | 37 (8.6%) | 53 (12.3%) * |
| IFN-α and IFN-ω | 30 (6.9%) ** | 36 (8.3%) |
| IFN-α alone | 5 (1.2%) | 8 (1.9%) |
| IFN-ω alone | 1 (0.2%) | 9 (2.1%) |

* AAN-I-IFNs-100 carriers

** AAN-I-IFNs-10 carriers
