# Supplementary material for: High nasopharyngeal SARS-CoV-2 load and delayed clearance in hospitalized patients with blood autoantibodies neutralizing type I interferons
Source: J Infect Dis. Author manuscript; Available in PMC 2026 Aug 12. (PMC13460755; doi:10.1093/infdis/jiag172)
Supplement: Supplementary file 10 [file NIHMS2185128-supplement-Table_S4.docx]

***Table S4:*** *Association between VLr and AAN-I-IFN status using linear models (with or without VLr < LoD values) and a left-censored Tobit model (considering VLr < LoD as censored values).*

| Model | Phenotype | N cases | N controls | Effect size (Standard Error) | Pvalue |
| --- | --- | --- | --- | --- | --- |
| Linear regression, VLr<LoD set to 1 | AAN-I-IFNs-10 | 23 | 261 | 1.17 (0.37) | 1.4x10^-3^ |
|  | AAN-I-IFNs-100 | 38 | 261 | 0.70 (0.30) | 0.018 |
| Linear regression, VLr<LoD excluded | AAN-I-IFNs-10 | 21 | 227 | 1.16 (0.36) | 1.3 x10^-3^ |
|  | AAN-I-IFNs-100 | 34 | 227 | 0.74 (0.29) | 0.011 |
| Left-censored Tobit regression | AAN-I-IFNs-10 | 23 | 261 | 1.23 (0.41) | 3.0 x10^-3^ |
|  | AAN-I-IFNs-100 | 38 | 261 | 0.74 (0.33) | 0.027 |
