# Supplementary material for: High nasopharyngeal SARS-CoV-2 load and delayed clearance in hospitalized patients with blood autoantibodies neutralizing type I interferons
Source: J Infect Dis. Author manuscript; Available in PMC 2026 Aug 12. (PMC13460755; doi:10.1093/infdis/jiag172)
Supplement: Supplementary file 11 [file NIHMS2185128-supplement-Table_S5.docx]

***Table S5:*** *Association between COVID-19 severity and infected cell loss rates or viral load at randomization between critical and non-critical patients.*

|  | Mean (±SEM) in critical patients | Mean (±SEM) in non-critical patients | Pvalue |
| --- | --- | --- | --- |
| Infected cell loss rate | 0.78 (±0.02) | 0.91 (±0.02) | 3.5x10^-4^ |
| Viral load at randomization | 3.48 (±0.14) | 3.01 (±0.14) | 0.012 |
