# Supplementary material for: High nasopharyngeal SARS-CoV-2 load and delayed clearance in hospitalized patients with blood autoantibodies neutralizing type I interferons
Source: J Infect Dis. Author manuscript; Available in PMC 2026 Aug 12. (PMC13460755; doi:10.1093/infdis/jiag172)
Supplement: Supplementary file 12 [file NIHMS2185128-supplement-Table_S6.docx]

***Table S6:*** *Association of AAN-I-IFNs status and* *empirical Bayes estimate of the infected cell loss rate (*$\eta_{\delta}$*) with COVID-19 severity (sample size: n=409), considering them separately or jointly in the adjusted logistic regression models.*

|  | Separate models * | | Full model ^$^ | |
| --- | --- | --- | --- | --- |
|  | Effect size (SE) | Pvalue | Effect size (SE) | Pvalue |
| AAN-I-IFNs-10 | 1.52 (0.50) | 6.3x10^-4^ | 1.39 (0.51) | 2.4x10^-3^ |
| Loss rate random effect | -1.01 (0.29) | 3.5x10^-4^ | -1.01 (0.30) | 7.1x10^-4^ |

* Separate models assessed the associations of AAN-I-IFNs status and, separately, the infected cell loss rate with COVID-19 severity, adjusting on age and sex.

^$^ The full model jointly assessed the association of AAN-I-IFNs and infected cell loss rate with COVID-19 severity, adjusting on age and sex.
