# Supplementary material for: High nasopharyngeal SARS-CoV-2 load and delayed clearance in hospitalized patients with blood autoantibodies neutralizing type I interferons
Source: J Infect Dis. Author manuscript; Available in PMC 2026 Aug 12. (PMC13460755; doi:10.1093/infdis/jiag172)
Supplement: Supplementary file 13 [file NIHMS2185128-supplement-Table_S7.docx]

***Table S7:*** *Association of AAN-I-IFNs status and VLr with COVID-19 severity (sample size: n=284), considering them separately or jointly in the adjusted logistic regression models.*

|  | Separate models * | | Full model ^$^ | |
| --- | --- | --- | --- | --- |
|  | Effect size (SE) | Pvalue | Effect size (SE) | Pvalue |
| AAN-I-IFNs-10 | 1.43 (0.57) | 4.9x10^-3^ | 1.24 (0.60) | 0.019 |
| VLr | 0.18 (0.07) | 0.012 | 0.17 (0.08) | 0.023 |

* Separate models assessed the associations of AAN-I-IFNs status and, separately, VLr with COVID-19 severity, adjusting on age and sex for AAN-I-IFNs and age, sex and TSO for VLr.

^$^ The full model jointly assessed the association of AAN-I-IFNs and infected cell loss rate with COVID-19 severity, adjusting on age, sex and TSO.
