# Supplementary material for: High nasopharyngeal SARS-CoV-2 load and delayed clearance in hospitalized patients with blood autoantibodies neutralizing type I interferons
Source: J Infect Dis. Author manuscript; Available in PMC 2026 Aug 12. (PMC13460755; doi:10.1093/infdis/jiag172)
Supplement: Supplementary file 15 [file NIHMS2185128-supplement-Fig_S2.docx]

***Figure S2:*** *Distribution of infected cell loss rate (δ, days^-1^), stratified by treatment arm and age.*
